# The influence of cattle breed on susceptibility to bovine tuberculosis in Ethiopia

**DOI:** 10.1016/j.cimid.2012.01.003

**Published:** 2012-05

**Authors:** Martin Vordermeier, Gobena Ameni, Stefan Berg, Richard Bishop, Brian D. Robertson, Abraham Aseffa, R. Glyn Hewinson, Douglas B. Young

**Affiliations:** a Animal Health and Veterinary Laboratories Agency, TB Research Group, New Haw, Addlestone, Surrey KT15 3NB, United Kingdom; b Aklilu Lemma Institute of Pathobiology, Addis Ababa University, PO Box 1176, Addis Ababa, Ethiopia; c International Livestock Research Institute, Biotechnology Department, P.O. Box 30709, Nairobi 00100, Kenya; d CMMI, Department of Medicine, Flowers Building, South Kensington, Imperial College London, SW7 2AZ, United States; f Armauer Hansen Research Institute, PO Box 1005, Addis Ababa, Ethiopia

**Keywords:** Zebu, Holstein–Friesian, Bovine tuberculosis, Susceptibility, Innate immune responses

## Abstract

Bovine tuberculosis in domestic livestock such as cattle is an economically important disease with zoonotic potential, particularly in countries with emerging economies. We discuss the findings of recent epidemiological and immunological studies conducted in Ethiopia on host susceptibility differences between native zebu and the exotic Holstein–Friesian cattle that are increasingly part of the Ethiopian National herd, due to the drive to increase milk yields. These findings support the hypothesis that native Zebu cattle are more resistant to bovine tuberculosis. We also summarise the results of experimental infections that support the epidemiological data, and of laboratory experiments that suggest a role for the innate immune response, and in particular interleukin-6, in the outcome of bovine tuberculosis infection.

## 1 Bovine tuberculosis in Africa and Ethiopia

Bovine tuberculosis (bTB) is a chronic bacterial disease of animals and humans. *Mycobacterium bovis* – the main but not exclusive causative agent of bTB – is a member of the *Mycobacterium tuberculosis* complex, which also comprises the important human pathogen *M. tuberculosis*, as well as *Mycobacterium canettii*, *Mycobacterium africanum*, *Mycobacterium pinnipedii*, *Mycobacterium microti*, and *Mycobacterium caprae*. These phylogenetically closely related bacteria share more than 99.9% chromosomal identity and they cause tuberculosis with similar pathology in various mammalian hosts [1].

In animals, bTB can affect a wide range of species including both domestic and wildlife species [2,3]. Amongst livestock the disease is most commonly associated with cattle and has a large economic significance. As a zoonotic disease, bTB is also a threat to public health where consumption of infected unpasteurised milk and other dairy products can be a source of human infection [4,5]. However, exposure to aerosols containing *M. bovis* are likely to also be an important risk factor for bTB in humans [6].

Prevalence data on bTB in cattle are generally scarce, but official data reported by member countries of the OIE (World Organization for Animal Health) suggest that the disease in domestic animals is widely distributed around the world and present on nearly all continents (e.g. [7,8]). Most industrialized countries have been successful in controlling or eradicating bTB from domestic animals by programmes that regulate animal movements or implement test-and-slaughter policies. However, bTB presents different problems in many developing countries, where poor or total lack of control policies has allowed the disease to persist or increase [7]. In addition, a surge in the semi-urban dairy industry in many developing countries has increased the risk of epidemic bTB. The majority of people in the developing world are directly dependent on their livestock for their livelihood, therefore a failure to control bTB is likely to have a negative impact on both their economy and health.

In sub-Saharan Africa, animal production is facing new challenges since demographic growth, urbanization, and economic development are all contributing to the increasing demand for milk, meat, eggs and other animal products [9,10]. The indigenous cattle and the prevailing extensive rural production system are unlikely to be able to satisfy the rise in demand for animal products, therefore intensification of animal husbandry is required. The combination of intensified animal husbandry and the development of peri-urban systems for livestock production have resulted in increased incidence of bTB. Nonetheless, over 85% of the African cattle population is raised in countries where bTB is not considered a notifiable disease and therefore not controlled [7].

Ethiopia, at the Horn of Africa, is home to the largest livestock population in Africa with over 50 million cattle, of which more than 99% are the indigenous Zebu breed; the remaining animals are of imported exotic or mixed breeds [11]. The first reports of bTB in Ethiopia date back to the mid 1970s when slaughterhouse studies confirmed the presence of the disease (reviewed in [12]). More recent studies in Ethiopia suggest that bTB is endemic in large parts of the country and that the prevalence varies depending on the husbandry system. In rural settings where cattle are grazed on pasture, both tuberculin testing and abattoir surveillance records a relatively low bTB prevalence ranging from 0.8 to 10 percent [13,14,15,16]. In contrast, on intensive farms dominated by exotic dairy cattle a much higher prevalence (24–34 percent animal prevalence) has been reported ([17] and Firdessa et al., 2011, in preparation). These farms are mainly situated in urban and peri-urban areas of the country, and supply milk to the city and town dwellers. These findings indicate a relatively low overall national prevalence of bTB in Ethiopia, but with localised hot-spots associated with intensive farming and imported exotic animals. Interventions to control the disease in these high-prevalence hot-spots will be crucial in preventing its future spread.

A significant share of the work presented in this review is the fruit of an extensive project that was funded by the Wellcome Trust (UK) under their “Animal health in the developing world” initiative. Most of the studies were undertaken in Ethiopia to investigate (1) the prevalence and epidemiology of bTB in domestic and wildlife animals as well as risk factors for zoonotic transmission, (2) genetic aspects of host susceptibility to bTB, (3) efficacy of BCG vaccination in cattle, and (4) impact of bTB on the Ethiopian economy. The outcome of this research, and several stakeholder meetings held in Addis Ababa on bTB, has clearly had a significant impact on the perception and interest in the subject amongst policymakers, researchers and concerned farmers.

## 2 Historical data on bovine tuberculosis susceptibility differences between cattle breeds

The natural trypano-tolerance of certain African *Bos taurus* (taurine) cattle in West Africa (e.g. N’Dama), in contrast to trypano-susceptible cattle breeds like Boran (i.e. *Bos indicus*, indicene, zebus), highlighted the fact that genetic traits in cattle can influence resistance to infectious diseases (reviewed in [18]). Bovine tropical Theileriosis (caused by the parasite *Theileria annulata*) provides another example, with Holsteins (*B. taurus*) being extremely susceptible and several *B. indicus* breeds (e.g. Kenana cattle from Southern Sudan) showing a degree of resistance [19].

Studies over the last 100 years have also indicated that differences exist in the relative susceptibility of different breeds of cattle to bTB. For example, Liston and Soparkar [20], and Soparkar [21] concluded that Indian *B. indicus* dairy cows were more resistant to experimental bTB than European, *B. taurus*, breeds. Although a more recent slaughterhouse study in India supported these findings that Indian breeds were less affected by bTB than pure European breeds like Jersey, Holstein, Friesians, Brown-Swiss [22], the earlier large-scale studies by Liston and Soparkar, and Soparkar [20,21] were ultimately and unavoidably flawed because the authors were unable to obtain and infect European breeds of cattle. This meant that they had to compare their results using Indian breeds, with data obtained much earlier in a different study using European breeds conducted in the UK [23]. Thus these studies lacked contemporaneous comparative control groups of European breeds.

## 3 Historical data of bTB susceptibility differences between African breeds

The incidence of bTB in East Africa, in particular in Uganda, in relation to cattle breeds, was studied by Carmichael in the 1930s [24]. Studying post-mortem statistics he concluded that the incidence of bTB was dramatically lower in Zebu cattle compared to taurine Ankole cattle (0.1–0.7%, compared to 12.5–41.4%; [24]). Based on these observations, he then conducted an experimental *M. bovis* challenge experiment using both Ankole and Zebu calves. His data confirmed his earlier epidemiological observations [25] as Zebu calves showed remarkable resistance compared to Ankole calves; Zebus showed a generalized infection initially after 90 days post-infection, that was contained and in some cases resolved over time (i.e. by 354 days) suggesting the development of protective immunity. In addition, differences between indicene and taurine breeds in the efficacy of BCG to protect against experimental bTB were reported by Ellwood and Waddington [26] who concluded that ‘the Zebu derived most protection from the [BCG] vaccination’.

In Ethiopia, PPD skin-testing of indicene and taurine cattle further supported the hypothesis that differences exist in their relative susceptibilities to bTB. For example, data collected in Eastern Shoa (central Ethiopia) indicates that diverse local *B. indicus* breeds had lower skin test prevalence (5.6%) compared to 86.4% in mainly Holsteins exotic breeds, with 13.9% prevalence in crosses (Tadelle, unpublished data). These studies did not compensate for the effect of animal husbandry conditions, although a recent study in Ethiopia [27] demonstrated that these are a major influence on the prevalence of bTB: cattle kept under high-intensity husbandry conditions (in-doors and at high population density) present with much higher skin test prevalence with markedly more severe disease, compared to cattle reared under low-intensity conditions on smallholder farms.

## 4 Results of our recent field studies

**Fig. 1:**
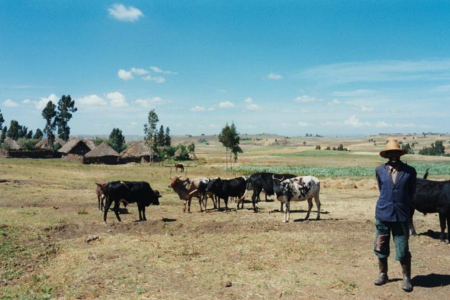
Typical smallholder herd in the study area (Selalle, North-Western Shoa region, Ethiopia). Shown are examples of Arsi zebus and Holstein-zebu crossbreeds. For example, the animal on the right is such a cross-bred animal. (Photo source: M. Vordermeier.)

**Fig. 2:**
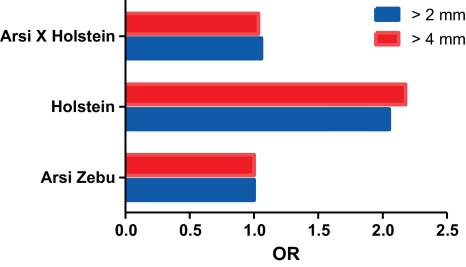
Breed differences in tuberculin skin test prevalence. Comparative tuberculin skin tests were applied to Arsi zebu, Holstein, and crossbreed animals, *n* = 1921, 925, 2578, respectively. Two interpretation criteria for the skin test were applied: reaction sizes of bovine tuberculin PPD minus avian PPD > 2 mm or > 4 mm. Data are expressed as Odds ratios. Data from Refs. [28,29].

To compensate for artefacts based on comparing cattle populations housed under different husbandry systems, our studies were focused on areas of Ethiopia where native zebu cattle (mainly the Arsi breed), Holstein cattle, as well as cross-breeds are kept under the same, low intensity management system. The study area chosen was North-western Shoa (Selalle) where animals are kept together on pasture under low intensity farming conditions. The study area contained around 6000 smallholders with average herd sizes of about 10 animals (Fig. 1). Half of these animals were of the Arsi Zebu breed, whilst the other half was composed of Holstein and Holstein-Zebu cross-breeds. About 10% of these herds were sampled and bTB prevalence measured using the single comparative tuberculin test, which compares reaction sizes after intradermal injection of avian and bovine tuberculin, PPD-A and PPD-B respectively. In total, 5424 cattle (925 Holsteins, 1921 cross-breeds and 2578 zebus) [28,29] were tested. In a parallel study, 153 skin test positive cattle were bought and examined post-mortem to establish their disease status (severity, lesion distribution) in relation to in vitro immune responses from whole blood cultures measured using interferon-gamma (IFN-γ) as a read-out. The results of the comparative skin test are obtained by measuring the difference in inflammatory reaction obtained in response to PPD-B minus PPD-A. A difference of ≥4 mm is the standard cut-off for a positive reaction and this showed significant breed differences comparing Holstein, Arsi zebus and cross-bred cattle, with about twice as many Holsteins testing positive, compared to Arsi zebus or cross-bred animals (Fig. 2; 22.2% in Holsteins compared to 11.6 and 11.9% in Arsi and crossbreeds, respectively; Odds ratios Holstein and crossbreeds compared to Arsi zebus: 2.18, 1.04, respectively [28]). We reassessed the interpretation criteria for the comparative intradermal tuberculin skin test in this Ethiopian cattle population using Receiver Operator Characteristics (ROC) analysis, which indicated that the cut-off value for a positive reaction (PPD-B minus PPD-A) should be ≥2 mm. However, even after applying this more sensitive cut-off, the higher skin test prevalence found in Holstein cattle was not significantly altered compared to either Arsi or cross-breeds (Fig. 2; skin test prevalence Holstein, Arsi, crosses = 24.9, 13.9, 14.6%, odds ratios of Holsteins and crosses compared to Arsis: 2.052, 1.058). This clearly indicates that there are significant differences in the susceptibility of indicene Arsi and taurine Holstein cattle, to bTB.

**Fig. 3:**
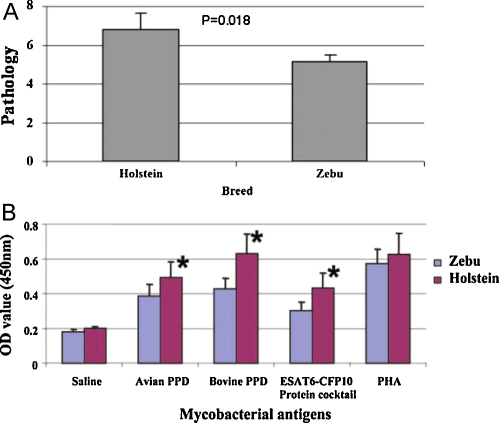
Pathology and IFN-gamma responses; a slaughterhouse study. (A) Mean pathology scores in skin test-positive cattle. Holstein, *n* = 50; Zebu, *n* = 73. Post-mortem analysis and scoring were performed as described in [27,30]. Figure from [28]. (B) IFN-γ responses to mycobacterial antigens in Holstein and zebu cattle after stimulation with avian and bovine PPD (used at 10 μg/ml), ESAT-6/CFP-10 proteins (each at 5 μg/ml) and PHA as positive control (5 μg/ml). IFN-γ as determined by BOVIGAM ELISA, data are expressed as mean ± standard error (from [27]).

In the same study, we were able to assess the pathology resulting from mycobacterial infection of cattle with respect to the extent and severity of visible pathology at post-mortem. This was assessed using a quantitative pathology scoring system developed for bTB vaccine research [30]. Interestingly, Arsi cattle presented with statistically significantly lower overall pathology compared to Holstein cattle (Fig. 3A [28]), although the differences were relatively small in absolute terms (mean pathology scores in Holsteins and Arsis, respectively: 6.84 and 5.21) [28]. Whilst no significant differences in the size of the skin test reactions were found in reactor animals of the two breeds [27], significantly lower IFN-γ responses to PPD-B, PPD-A and a cocktail of the *M. bovis*-specific antigens ESAT-6 and CFP-10 [31,32] could be demonstrated in Arsi zebu cattle compared to Holstein cattle (Fig. 3B [27]). This is perhaps not surprising as it has been shown that responses to ESAT-6 correlated positively with the degree of pathology [30]. An interesting additional observation was made when the causative agents of bTB in these cattle were identified, by culture of mycobacterial species from tissues collected at post-mortem. Apart from *M. bovis*, the organism commonly associated with bTB, *M. tuberculosis* as well as non-tuberculosis-complex strains such as *Mycobacterium avium avium* were also found in tissue lesions [33]. No differences were observed in the mycobacterial species causing bTB in indicene compared to taurine cattle.

## 5 Experimental *M. bovis* infection studies of taurine and indicene cattle

**Fig. 4:**
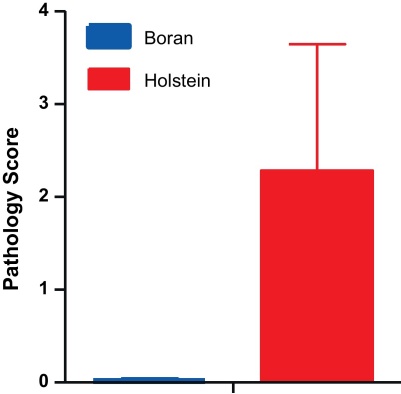
Results of experimental *M. bovis* infection. Groups of 6 Boran and 6 Holstein calves (around 6 months old) were infected intratracheally with a UK *M. bovis* isolate (AF2122/97 [43]). Post-mortem examinations were performed after 4 months and pathology was scored as described [30]. Data are expressed as mean total pathology scores ± standard errors.

To confirm experimentally the epidemiological data described above, a pilot experiment was conducted to test the response of taurine compared to indicene cattle to *M. bovis* infection. As it was logistically difficult to source Arsi zebus and conduct the experiment in Ethiopia, this experiment was conducted in South Africa using Holstein and Boran zebu cattle. Borans were selected because they are genetically closely related to Arsi zebus. Groups of six Boran and six Holstein–Friesian calves were each infected with a low dose of *M. bovis* and kept for 16 weeks, after which they were euthanized and their disease status established by post-mortem examination. The very low infective dose used [34] meant only half of the Holstein calves showed visible pathology, which is typical of bTB. In contrast, none of the similarly infected Boran calves showed any visible pathology, an outcome that was also reflected by the pathology scores (Fig. 4). This pilot experiment provided confirmatory evidence that native African indicene cattle breeds are indeed more resistant to bTb than exotic taurine breeds, such as Holstein–Friesians.

## 6 Investigation of potential immunological parameters underlying this enhanced resistance

**Fig. 5:**
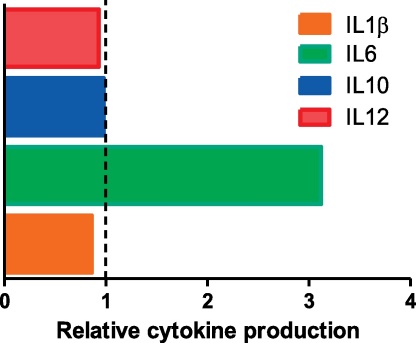
Innate responses: Cytokine production in Holstein CD14 monocytes infected with virulent mycobacteria (*M. bovis* or *M. tuberculosis*, MOI =2) relative to responses of monocytes isolated from Sahiwal zebus (horizontal line = responses observed with zebu monocytes). Cytokine responses in culture supernatants were determined using a bovine cytokine/chemokine multiplex system [44]. Data from [37].

As initially proposed to account for trypano-tolerance, we hypothesised that the higher resistance of zebu cattle to bTB was either based on innate or acquired immune processes, or on non-immune mechanisms [18]. Initially, we compared the antigen specificities of B and T cells isolated from naturally infected (skin test positive) Holstein and Arsi zebu cattle. PBMC were prepared and tested by IFN-γ ELISPOT, and antibody responses were probed by multi-antigen print immunoassay (MAPIA [35]). In addition, we also determined IL-10 levels in PBMC culture supernatants. T cells from both sets of animals recognised the same antigens in the ELISPOT assays, with *M. bovis*/*M. tuberculosis* HSP65, CFP-10, MPB83, Ag85A and Rv3979c the most frequently recognised antigens (Cockle, Ameni, Vordermeier, unpublished data). Serum antibody responses were directed towards MPB83 and MPB70, which are generally considered to be the sero-dominant antigens recognised after bTB infection of cattle [36]. In addition, both sets of animals produced comparable amounts of IL-10 after PPD-B stimulation (Cockle, Ameni, Vordermeier, unpublished data). Thus, in these investigations we could not demonstrate breed differences in the adaptive immune responses of infected cattle that would account for either the lower IFN-γ responses demonstrated in Arsi cattle, or why Arsi cattle seem to be less susceptible to bTB. As further studies of the immune response were logistically difficult in Ethiopia, we decided to investigate innate immune responses by infecting CD14^+^ monocytes isolated from uninfected Holstein or Sahiwal cattle. These studies were undertaken in the UK using blood from Sahiwal zebus provided by Professor Glass (Edinburgh University, UK). Monocytes were infected with either live *M. bovis* or *M. tuberculosis* at an MOI = 2, and a range of bovine cytokines and chemokines were measured in the culture supernatants. Nitric oxide production post-infection was also determined. We found no differences in the post-infection production of IL-1ß, IL-10 and IL-12 between Holstein or Sahiwal monocytes ([37]; Fig. 5, relative cytokine levels in supernatants from Holstein cows relative to Sahiwal monocytes of 0.86 and 0.98). In contrast, Sahiwal monocytes produced significantly less IL-6 (Fig. 5, relative IL-6 levels in Holsteins compared to Sahiwal monocyte cultures: 3.12) [37]. Interleukin-6 is a pleiotropic pro-inflammatory cytokine produced by innate immune cells as well as T-cells. Studies in a number of species have evaluated the role of IL-6 in tuberculosis, however conflicting data can be found when attempting to ascertain whether IL-6 exerts beneficial or detrimental effects in tuberculosis. For example, studies in IL-6 gene-disrupted mice suggest a role in protection against tuberculosis [38]. In contrast, some human studies have shown an association between IL-6 and active pulmonary TB, compared to healthy subjects [39,40]. Several studies have used Single-Nucleotide Polymorphism (SNP) patterns to evaluate pre-disposition to tuberculosis and found a positive relationship between a SNP associated with high IL-6 production (position – 174 of the gene) and the relatively high rate of tuberculosis amongst a Canadian aboriginal population [41]. These latter studies therefore support our data of differential IL-6 production by infected macrophages from different cattle breeds, associated with the virulence difference suggested by our previous studies. However, further studies are needed to investigate this in particular using different innate cells such as dendritic cells, innate cells isolated from Arsi cattle, and testing the survival or killing of *M. bovis* inside innate immune cells.

## 7 Study of genetic factors influencing susceptibility to bTB

Interestingly, a recent preliminary study assessed genetic factors influencing susceptibility to bTB in the British cattle herd (which are all of taurine origin) [42]. This analysis was based on a candidate gene approach using microsatellite typing, and assessed the association of genotype and tuberculin skin test positivity. They found several associations, with one of the most strongly associated loci being proximal to a region containing genes for IFNGR1, IL20RA, and IL22RA2, genes of obvious immunological relevance [42]. To find genetic factors associated with skin test reactivity in a study of relatively closely related taurine breeds is therefore encouraging, and in our field studies in Ethiopia we also collected samples to determine potential genetic associations with the higher resistance in Arsi cattle. Our approach is based on admixture mapping using bovine SNP microarray analysis and samples collected predominantly from Holstein-Arsi cross breeds, and analysis is still on-going (D. Bradley, Y. Hirtu et al., unpublished data). The genetic factors underlying resistance and susceptibility to infectious diseases of livestock such as Theileriosis can be multi-factorial [18] and could include genes in involved in innate, acquired immunity of indeed non-immune parameters. It is highly likely that similar multi-factorial gene profiles will account for the suggested susceptibility difference of zebu and Holstein cattle.

## 8 Concluding remarks

Our extensive field studies have provided epidemiological evidence that at least some zebu breeds are less susceptible to bTB and, if infected, present with less pathology than exotic breeds such as Holstein–Friesians. These results were supported by a small experimental infection study and by pilot studies on innate and acquired immune mechanisms that start to address the mechanisms underlying the observed differences in breed susceptibility to bTB. Apart from the obvious scientific aspects, the importance of these findings are of practical relevance, as they highlight the potential risks from increasing milk yield through the introduction of exotic cattle breeds that potentially are more susceptible to bTB then native zebus animals. Although the extent and risk of human zoonotic *M. bovis* infections are unclear, the fact that *M. tuberculosis* could be isolated from a sizable proportion of tuberculous cattle in our study area, could indicate a potential cattle-to-human transmission risk for *M. tuberculosis.*
